# Nurturing nursing in India: need for governance reform

**DOI:** 10.1186/1753-6561-6-S5-O32

**Published:** 2012-09-28

**Authors:** Kabir Sheikh, VR Raman, Kaveri Mayra

**Affiliations:** 1 Public Health Foundation of India, New Delhi, India

## Introduction

Recent Universal Health Coverage recommendations in India have been developed on the presumption of a principal role of nurses as the backbone of public health services. Yet this sector faces a distinct and formidable set of challenges. Underproduction and migration underpin shortages in the number of nurses per capita. The quality of nursing care is hampered by poor standards of education. Nurses also face tribulations in the form of discrimination, harassment and subordination (to doctors) in the health administration. The solutions for these interwoven challenges are located in more effective governance of the nursing sector.

## Methods

We undertook a review of the literature to characterize the state of nursing governance in India, and identify the challenges for effective governance. Keyword searches were conducted on academic search engines and websites of internet booksellers for peer-reviewed literature, and on general search engines for relevant research reports, case studies and commentaries, supplemented by physical search for sources in the library of the Public Health Foundation of India. The resulting index of books, articles and academic material was organized by theme, and the literature review was written up in essay style.

## Findings

The challenges to effective governance of nursing identified include a span of structural, operational and political factors. Notable among these are the lack of a well-articulated policy framework for human resource development and management for nursing in the government sector. As the largest human resource pool within the health system, the nursing cadre necessitates institutional arrangements for effective supervision, skill development and career advancement, which are inadequate or absent in most states and at national level.

Typifying these administrative failures is the absence of a focal body for nursing governance in the Ministry of Health & Family Welfare and poor representation of nurses in key decision-making offices. The capacity of public services to retain and support nurses is constrained variously by poor working conditions and outdated personnel remuneration and norms. These challenges are also mirrored in the private sector, where they are amplified by the absence of credible regulation of either the performance of, or welfare measures for nurses in employ. Finally, effective professional governance, through nursing councils, is thwarted by weak institutional arrangements, antiquated laws, insufficient representation of midwives in the national council, conflicted relationships between state and national councils, and the dominating influence of medical professional bodies.

## Discussion

Even as more effective governance is required to address the triple challenge of nursing adequacy, workforce and welfare, systems for governance of nursing in India face several challenges, partly reflecting those confronting the sector at large. This suggests that multipronged reforms to the architecture of nursing governance are warranted before implementing wider sectoral reforms. World Health Organization’s conceptual framework for equitable access to quality nursing and midwifery care presents a credible framework for the adoption of governance reforms. The framework identifies three pillars of nursing governance, i.e. policy and planning, education and development, and deployment and utilization, respectively denoting the institutions that need to be strengthened and developed.

The relative paucity of systematic knowledge on shortfalls in nursing governance is also remarkable, which highlights the need for further research. In particular, empirical analyses of the architecture and performance of governing institutions can help to identify specific targets and priorities for governance reform.

## Competing interests

Authors declare that they have no conflict of interest.

## Funding statement

This study was funded by the Oxfam India.

